# Investigating selection on viruses: a statistical alignment approach

**DOI:** 10.1186/1471-2105-9-304

**Published:** 2008-07-10

**Authors:** Saskia de Groot, Thomas Mailund, Gerton Lunter, Jotun Hein

**Affiliations:** 1 Department of Statistics, University of Oxford, 1 South Parks Road, OX1 3TG, UK; 2 BiRC affliation: Bioinformatics Research Center, University of Aarhus, Hoeg-Guldbergsgade 90, 8000 Aarhus, Denmark; 3 MRC Functional Genetics Unit, Department of Physiology, Anatomy & Genetics, University of Oxford, 1 South Parks Road, Oxford OX1 3QX, UK

## Abstract

**Background:**

Two problems complicate the study of selection in viral genomes: Firstly, the presence of genes in overlapping reading frames implies that selection in one reading frame can bias our estimates of neutral mutation rates in another reading frame. Secondly, the high mutation rates we are likely to encounter complicate the inference of a reliable alignment of genomes. To address these issues, we develop a model that explicitly models selection in overlapping reading frames. We then integrate this model into a statistical alignment framework, enabling us to estimate selection while explicitly dealing with the uncertainty of individual alignments. We show that in this way we obtain un-biased selection parameters for different genomic regions of interest, and can improve in accuracy compared to using a fixed alignment.

**Results:**

We run a series of simulation studies to gauge how well we do in selection estimation, especially in comparison to the use of a fixed alignment. We show that the standard practice of using a ClustalW alignment can lead to considerable biases and that estimation accuracy increases substantially when explicitly integrating over the uncertainty in inferred alignments. We even manage to compete favourably for general evolutionary distances with an alignment produced by GenAl. We subsequently run our method on HIV2 and Hepatitis B sequences.

**Conclusion:**

We propose that marginalizing over all alignments, as opposed to using a fixed one, should be considered in any parametric inference from divergent sequence data for which the alignments are not known with certainty. Moreover, we discover in HIV2 that double coding regions appear to be under less stringent selection than single coding ones. Additionally, there appears to be evidence for differential selection, where one overlapping reading frame is under positive and the other under negative selection.

## Background

In the past few years we have witnessed an explosion in the viral genomic data available. GenBank alone holds over 80,000 close to complete viral genomes, and numbers are rising fast. For example, since the submission of the first SARS genome in May 2003, over 140 more have been published. With this genomic data at hand we hope to finally be able to tackle our understanding of viruses. Mechanisms of selection, that is to say the rate *f *at which a mutation resulting in a change in amino acid is accepted, and evolution on viruses are still strongly debated, and a methodology which is trimmed towards answering these questions is required. A step towards this is our attempt to develop a method which can deal with the vast amount of viral data, as well as the complexity of viral genomes and their high divergence and subsequent unreliability of alignment.

Several papers [1,4,5,7,13-16,18] have been dedicated towards the study of selection on viral genomes, in particular focusing attention on the evolutionary behaviour of overlapping reading frames. These are a feature common to viruses, where due to the three periodicity of the genetic code, up to three genes may be encoded simultaneously. The constraints placed on a nucleotide involved in such a multiple coding region will naturally have an effect on its mutational pattern, and as a result the concept of selection is complicated further. Another complication is the uncertainty of alignments when dealing with genomes of reasonable evolutionary distance. Recent papers have shown that parameter estimation can be greatly biased by the use of a fixed alignment [10].

It is often thought that overlapping regions tend to be more constrained in their evolution than single coding ones, since a mutation may cause a non-synonymous substitution in up to three genes simultaneously. Some methods rely on these assumptions for the *de novo *detection of overlapping genes [19,22].

Various researchers have attempted to measure selection acting on overlapping reading frames, by investigating the *K*_*a*_*/K*_*s *_ratio within these regions for seperate reading frames [4,7,14-16]. Comparing non-synonymous to synonymous substitution rates only makes sense when the synonymous substitutions are unconstrained. In the case of coding for multiple genes, however, a synonymous substitution in one gene may well be non-synonymous in the other and thus constrained. This biases the analysis towards an under-estimation of the 'true' synonymous substitution rate and thus can lead to the false inference of positive selection.

An attempt to resolve this problem has been made, for example by focusing on synonymous substitutions in one reading frame which indeed are unconstrained in the other [18]. Hein & Støvlbæk [5] developed an evolutionary model particular to multiple coding regions, and used this for a study of selection on these. de Groot *et al*. [1] used this model of varying selection to comparatively annotate two viral genomes with evolved gene structure. McCauley *et al*. [13] incorporated a slightly extended version into their multiple sequence annotation method, which additionally provides a selection annotation of the genome. However, their method looks at selection on an individual nucleotide level, and does not make assumptions about the modelling of selection on specific regions.

Our method presented here looks at selection on genomic segments as opposed to nucleotides, and thus in overlapping coding regions can discern selection for different reading frames. We may therefore attempt to draw conclusions about the nature of not only selection but also the interaction of selection on two different genes. Also, to study the imprint of evolution on viral genomes, it is necessary for the samples to have a reasonably high level of divergence. A benchmark herefore in our experience would be an evolutionary distance *a *+ 2*b *of at least 0.4. Since more divergent genomes are harder to align, this brings uncertainty about the alignment into the inference. We decide to circumvent this problem by considering the set of *all *possible alignments – and their corresponding likelihood under our model – , as opposed to a fixed 'optimal' alignment. This method has previously been used for similar purposes, to minimize variability in parameter estimation due to uncertain alignments [10,12].

We work with a simple indel model, together with our evolutionary model, to generate a pairwise statistical alignment. For two sequences *x *and *y*, a set of seed parameters then gives us the probability *p*_*ij *_of each *i*^*th *^position *x*_*i *_being aligned with each *j*^*th *^position *y*_*j*_. We subsequently work with expected observations as opposed to actual ones. We iteratively calculate the alignment probabilities and the maximum likelihood estimates of evolutionary parameters, until we reach a given level of convergence. We also extend our methodology to a multiple pairwise method.

The work presented in this paper thus improves on both the above methods [1,13] by our ability to pry apart selection for two genes on overlapping segments and us not having to rely on a fixed alignment anymore.

We run a simulation study to gauge the improvement made by considering all possible alignments as opposed to a single fixed one. Even though viruses containing a large number of multiple coding sites might be expected to be easy to align, our simulation results suggest that this is not necessarily the case. The improvement in parameter estimation made by getting rid of uncertainty in alignments appears to be non-negligible, even for viruses with overlapping reading frames.

We run our method on a set of 5 HIV2 sequences, as well as a set of 3 Hepatitis B genomes. These are good candidates for analysis of overlapping reading frames, with 11% of the HIV2 genome being double coding and an average overlapping segment being of length 171 nucleotides. Hepatitis B is even more compact with 49% of the genome being double coding and an average overlapping segment length of 532 nucleotides. We subsequently investigate various questions relating to overlapping reading frames and the selectional mechanism underlying these.

## Results

### Simulation

We test our method on simulated data, to see whether summing over all alignments does actually improve results notably. All the results in this section, unless stated otherwise, are obtained using the 'worst-case-scenario' of only two sequences.

By taking a 600 nucleotide sequence chunk out of a double coding region of the Hepatitis B NC00397 sequence, we construct a long double coding region, flanked by 300 nucleotides on either side of background sequence. We let this evolve according to the TKF91 model [21] into a descendent sequence, where the Match-Match state emits a descendant according to the Hein & Støvlbæk [5] model with specified evolutionary parameters. We use a gap opening probability of 0.02 and a gap extension probability of 0.4 – these being values similar to the ones encountered in the real sequences we wish to analyse. We also only allow gaps of length 3 within coding regions, so as not to cause a frame shift in coding. We fix all selection parameters to 0.5 and test a variety of evolutionary distances, with transition rate *a *ranging from 0.2 to 0.7 and transversion rate *b *= *a*/2.

**Figure 1:**
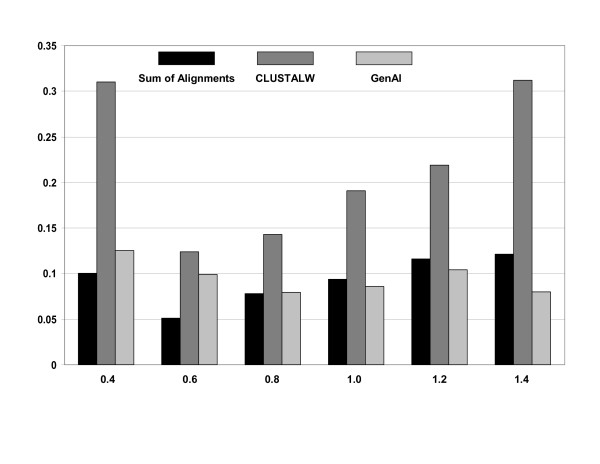
**Simulation Results: Varying Evolutionary Distance**. Simulation results between two sequences for a double coding region of length 600 of varying evolutionary distance. The figure plots the average estimation error of the statistical method and the fixed alignment method using both ClustalW and GenAl, versus the evolutionary distance between the two sequences. The estimation error is measured as the fraction of the average absolute deviation to the true parameter value and the true value itself. The evolutionary distance is measured as *a *+ 2*b*, where *a *and *b *are transition and transversion rates respectively.

We annotate using our statistical alignment method described above, as well as performing parameter optimization on a fixed alignment produced by both GenAl [6] and ClustalW[20]. As we can see from Figure 1, ClustalW gives consistently rather bad results, since it is not designed to deal with overlapping coding regions. Our method achieves better results than GenAl on sequences of evolutionary distance less than 0.8, but cannot quite compete with GenAl on sequences further apart. Here our estimation error is shown as the fraction between the average absolute deviation of our estimated parameters to the true parameter value and the true value itself.

The statistical alignment method performs, when applied to evolutionary distances we are realistically going to encounter, within 10% of the true value. Similar results hold for a number of other tested scenarios, including cases where one reading frame is under much stronger selection than the other and both are under positive or both under strong negative selection.

**Figure 2:**
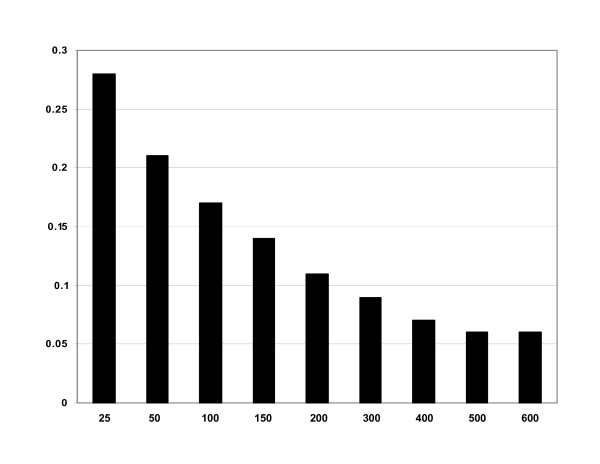
**Simulation Results: Varying Gene Length**. Simulation results between two sequences for a double coding region of varying length nested in a single coding region of length 800. The figure plots the average estimation error of the statistical method, versus the gene length of the double coding region. The estimation error is measured as the fraction of the average absolute deviation to the true parameter value and the true value itself.

We wish to find out what effect the length of a double coding region has on our estimation accuracy. Letting the length of the double coding region in our above simulation vary from 600 down to 25, with transition and transversion rate 0.4 and 0.2 respectively, we obtain Figure 2. As to be expected, the shorter the region, the worse our prediction results, since our data set decreases. However, above a length of 50 nucleotides we start picking up selection within a distance of ± 0.15, and above 200 nucleotides we are within the ± 0.1 mark. This is reassuring, since as mentioned above the average double coding region in HIV2 and Hepatitis B is 171 and 532 respectively.

We test the confidence levels of our predictions, trying to create as 'realistic' simulated data as possible. In the light of our real data analysis, we take the Hepatitis B genome NC00397 and split it into 7 different regions, a new one starting whenever there is a change in gene structure. We evolve the sequence according to our indel model with varying transition and transversion rate of *a *= 0.2 – 0.8 and *b *= *a/*2 respectively, and fixed selection strength of 0.5 for each of the different regions. Depending on the evolutionary distance and closely related to our results in Figure 1, we achieve an accuracy of approximately 70 – 94% with both the statistical alignment method as well as the fixed alignment method using GenAl, versus 20 – 72% for the fixed alignment method using ClustalW. In contrast using the true alignment gives us an accuracy of 78 – 96%. Here our estimate is counted as correct if the true value lies within the error bars around the estimated value. This is naturally highly dependent on the width of our error bars, which in some cases are indeed large, simply due to lack of data. However, the error bars for the parameter estimates of both the fixed and the summed alignment are close to identical, and thus the measure is valid if only for the sake of direct comparison.

One of the reasons for the comparatively low performance on ClustalW alignments might be that those alignments often do not conserve the reading frame. As we wish to make as fair a comparison as possible, we therefore additionally manually adjust alignments to be more 'reasonable' by adjusting gap placement to conserve the reading frame. This does indeed result in considerable inprovement, thus demonstrating the volatility of results when dependent on one particular alignment. However, even when improving the fixed ClustalW alignment, the resulting accuracy after manual adjustment still falls short of that achieved by the statistical alignment method, reaching only 40 – 70%.

Finally, we compare our results on the last setup using simulated descendants of the Hepatitis B genome in a pairwise versus a multiple sequence scenario. When adding up to four sequences, we observe the error bars getting notably tighter and simultaneously our estimation error decreasing by about 0.01 per added sequence. This implies, as desired, a more precise estimation of selection factors for multiple sequences.

### Hepatitis B

**Figure 3:**
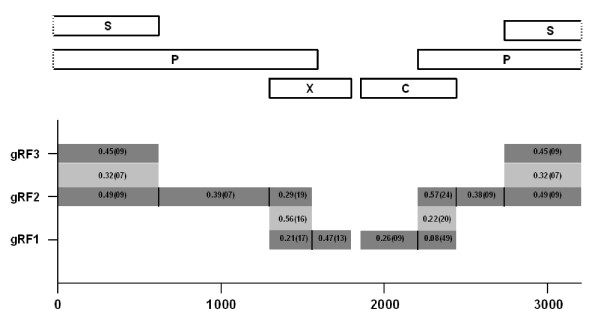
**Hepatitis B Annotation**. The estimated parameters for the seven different genomic regions of the Hepatitis B virus NC003977 based on the Woodchuck Hepatitis genome J02442 and the Ground Squirrel Hepatitis genome K02715. Here the darker shades refer to the selection acting on one gene only, and the lighter shades are the selection factors for non-synonymous substitutions in both genes. The error in the parameter estimates (one standard deviation) is given in brackets, in units of 10^-2^. The x-axis delineates the position on the genome.

We run our method on the Hepatitis B strand NC003977 and 'descendants' Woodchuck Hepatitis B strand J02442 and Ground Squirrel Hepatitis K02715, with sequences and gene structure downloaded from GenBank. As seed parameters we have all values set to 0.5 and wait between iterations for a difference in our loglikelihood of *<*1. Our method takes ~40 seconds to reach convergence and results are shown in Figure 3.

To see how a region acts when viewed as a whole, we also calculate the average selection acting on double coding regions, by weighting the expected counts for each mutation by the appropriate selection coefficient – in the case of a single non-synonymous change in gene *A *or *B *by the factor *f*_*A *_and *f*_*B *_respectively, and in the case of two non-synonymous changes by the joint factor *f*_*AB*_. Table 1 shows the values obtained for the different regions, both single and double coding. We can see that when viewed like this, the double coding regions are on average under 0.41 selection, and thus not greatly different to the single coding ones at an average of 0.39.

**Table 1:** The average selection acting on the Hepatitis B genome.

Region	Genes	Type	Selection
1	C	Single	0.26
2	C,P	Double	0.31
3	P	Single	0.38
4	P,S	Double	0.40
5	P	Single	0.39
6	P,X	Double	0.46
7	X	Single	0.47

The average selection acting on each of the seven regions of the Hepatitis B genome, measured by weighing each expected mutation by its appropriate selection coefficient. Selection on double coding regions appears to tend to be more lenient

Due to more than 1500 sites in the Hepatitis B genome being multiple coding, we may reasonably test whether the simpler multiplicative model is an equally good fit to the full one used above. Setting *f*_*AB *_= *f*_*A*_·*f*_*B *_we may perform a likelihood ratio test between the full and the restricted model, where selection acting on two different genes simultaneously gets multiplied up. With -2log Λ = 18 for 3 added parameters, the full model fits the date significantly better than the restricted multiplicative one (*P *= 0.0004).

### HIV2

**Figure 4:**
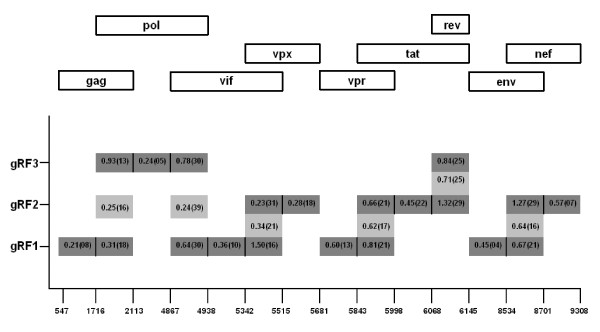
**HIV2 Annotation**. The estimated parameters for the seven different genomic regions of the HIV2 J04542 genome based on the HIV2 genomes U27200, M30502, DQ307022 and M15390. Here the darker shades refer to the selection acting on one gene only, and the lighter shades are the selection factors for non-synonymous substitutions in both genes. The error in the parameter estimates (one standard deviation) is given in brackets, in units of 10^-2^. The x-axis delineates the position on the genome (not to scale).

We apply our method to the HIV2 genomes J04542 with reasonably diverged 'descendants' U27200, M15390, DQ00835 and M30502, by splitting the genome into different regions whenever there is a change in gene structure. Setting all our initial parameters to 0.5, as above, we obtain a selection annotation for the different regions. The results of our parameter estimates are given in Figure 4.

As we can see, there is a marked difference between the estimated selection strengths underlying the different regions, with selection ranging from 0.21 – 1.50. Our results seem to suggest that genes encoded by double-coding regions often show contrasting modes of evolution, where one gene is highly conserved, whereas the other is less so. For example, in the second gene from the left, the *pol *gene, we see the middle section being under rather stringent selection of 0.24, whereas the two flanking regions are under less negative selection of 0.93 and 0.78. The respective overlapping sections in the other reading frames are under selection of 0.31 and 0.64. Similarly with the latter section of the following *vif *gene, we can see a dramatic increase in positive selection acting on the overlapping region, which rises to 1.50 against a selection of 0.23 in the other reading frame. Naturally all these estimates are made on relatively small regions, and thus have relatively large error bars, but tendencies towards a distinction between fast and slow evolving overlaps are nonetheless demonstrated. On the other hand, the selection on the overlap between the fifth and sixth gene in line – the *vpr *and the *tat *gene – is close to equal in both reading frames, thus indicating that the otherwise observed high and low selection values are not mere inevitable artefacts of our model. We return to this in the discussion.

One of the most remarkable observations is that within each reading frame, selection on single coding regions appears to be *more *constrained than in double coding ones. As before, we calculate the selection acting on each region as a whole, as shown in Table 2 and see that on average the single coding regions are under selection of strength 0.39 whereas the double coding regions seem to be under less stringent selection of an average of 0.64. This is in line with the results shown by de Oliveira *et al*. [2] and more recently by McCauley *et al*. [13], but somewhat contrary to general belief [19,22]. Clearly within the HIV2 genome there is much less data than with Hepatitis B, so it is harder to assign a true significance to these figures. However, our results do appear to suggest less stringent selection on overlapping regions than on single coding ones, thus maybe indicating the overlapping regions to be a relatively young feature in the virus.

**Table 2:** The average selection acting on the HIV2 genome.

Region	Genes	Type	Selection
1	gag	Single	0.21
2	gag, pol	Double	0.51
3	pol	Single	0.24
4	pol, vif	Double	0.54
5	vif	Single	0.36
6	vif, vpx	Double	0.63
7	vpx	Single	0.28
8	vpr	Single	0.60
9	vpr, tat	Double	0.44
10	tat	Single	0.45
11	tat, rev	Double	0.70
12	env	Single	0.45
13	env, nef	Double	0.99
14	nef	Single	0.57

The average selection acting on each of the seven regions of the HIV2 genome, measured by weighing each expected mutation by its appropriate selection coefficient. Selection on double coding regions appears to tend to be more lenient

## Discussion

We have introduced a novel method for estimation of selection strengths that explicitly incorporates uncertainty in estimated alignments. By integrating a statistical alignment procedure into our parameter estimation, we do not rely on a fixed alignment input. Instead, we calculate the expected number of observations, and are thus weighting our parameter estimates by the probability of each possible alignment. We naturally can not compete with knowing the true alignment, something which sufficient and extremely time consuming manual work can get close to. We do however offer a fast, automatic and efficient alternative to the use of a fixed alignment, which provides a quick and easy way for producing selection factors for different regions in a viral genome. We outperform alignments given by ClustalW consistently. We even beat GenAl for sequences of evolutionary distance below 0.8 and only do slightly worse for ones further apart. It is however additionally worth noting that the sequences we have and generally will be dealing with, will generally have an evolutionary distance of 0.4 – 0.9. We are therefore encouraged to see that our method is competetive compared to the slightly more refined GenAl and hope that this is amplified once also extended to include protein alignment. More importantly our method is statistical, which means it can be more readily incorporated into a maximum likelihood estimation framework, whereas GenAl works on a count-basis.

We test our method in a number of different simulation studies against the use of a fixed alignment, which we obtain using ClustalW. We show that on average our statistical approach has up to 30% higher absolute sensitivity, and that both evolutionary distance and the length of a double coding region have a lesser effect on our results than when using a fixed alignment.

Our study focuses on trying to understand the selection mechanism underlying overlapping reading frames. On the Hepatitis B genome, which boasts over 1500 multiple coding sites, we investigate several questions such as the selection a mutation is under, when it causes a non-synonymous mutation in two genes simultaneously. That is to say, if gene *A *and gene *B *are under selection *f*_*A *_and *f*_*B *_respectively, will a mutation affecting both necessarily be under selection *f*_*A*_·*f*_*B*_? A likelihood ratio test between the restricted multiplicative and the full model suggests this is not the case.

We also investigate the strength of selection on double coding regions, with different genomes indicating different results. In Hepatitis B we notice selection on double coding regions not being significantly different to that acting on single coding regions. In HIV2 however, surprisingly, single coding regions appear to be on average under up to two-thirds as stringent selection as double coding regions, supporting the findings of de Oliveira *et al*. [2], McCauley *et al*. [13], but not of Spiropoulou & Nichol [19], Walewski *et al*. [22].

Another feature which is particular to our method, is that we may seperate selection acting on the different reading frames in an overlapping region. We find especially in HIV2 a certain division of selection occurring, similar to that observed in Potato Leafroll Virus [4] and in Microviridae [16]. Essentially, it appears as though in an overlapping region one gene can take over the fastly evolving function, whilst the other behaves more conserved. Since this is not something we observed in our simulation studies, it seems to be no artefact of our model.

One possible evolutionary scenario that could explain this observation is the following: when an overlapping region is 'created'for example by the elongation of one of the genes involved, then it is likely to initially be under non-negative selection. Since the organism survived both with and without the overlap, it might be expected to be able to evolve without detrimental effects. A thus possible behaviour would be for the newly coding region to be encouraged to evolve quickly, whilst the other gene remains under negative selection as before. The estimated selection strengths may subsequently help deduce which overlaps are the 'newer' regions – for example our study suggests that the *pol *gene extended itself both onto the *gag *and the *vif *gene. The effect would essentially be similar to that noted on selection occurring on duplicated genes, where the duplicated gene reaches fixation in the population due to initially being under positive selection [24].

Up till now, other methods dealing with related issues have made use of the concept of *K*_*a*_*/K*_*s *_ratio, which however creates problems when applied to overlapping reading frames [4,7,14-16]. For this reason McCauley *et al*. [13] decided on a different HMM based approach and estimated selection as acting on a single nucleotide basis, but at the cost of not being able to pry apart selection acting on different reading frames. Most importantly however, all of the above methods use a fixed alignment and are thus prone to a great variability in their estimated parameters, dependent on the alignment. Our method manages to circumvent this problem by using a statistical approach, and thus we account for the uncertainty inherent in the alignment by considering all, rather than picking a single "best" alignment. The improvement we observe by doing this, makes us suggest that our approach of marginalizing over alignments may benefit other sequence-based inferential methods, such as for example methods for identifying conserved binding motifs.

One drawback to our method is the fact that for each descendent sequence we model transition and transversion rates as constant along the genome. This is a gross simplification, and something that should be dealt with in future work. As mentioned above, we would also like to superimpose protein alignment on our existent statistical alignment method, in accordance with the idea behind [6]. Another point is our fixing a partition prior to analysis. It would be even more interesting to be able to incorporate a hidden Markov model approach, in which breakpoints between regions would be chosen organically from the data. We could then truly start questioning which parts of the genome behave in different ways, as opposed to being restricted to the 'trial and error' approach that is the essence of our method now.

## Methods

### Outline

**Figure 5:**
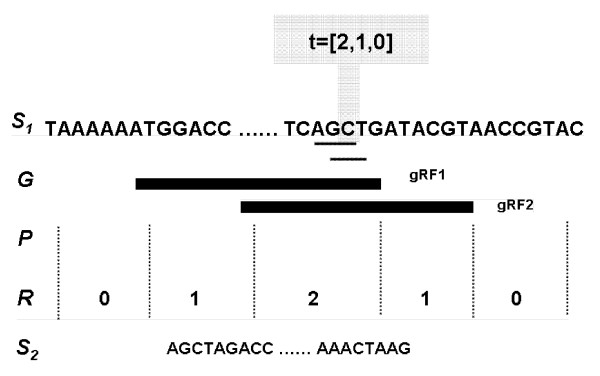
**Sequence Annotation**. An example of our input data and annotation. We see here the 'ancestral' sequence *S*_1_, whose genes structure *G *is given by coding regions in two reading frames GRF1 and GRF2. We apply a partition *P *to the sequence, where a breakpoint occurs whenever there is a change in gene structure. We annotate this partition with *R *= 3 different types of region for non-, single and double-coding respectively. Finally we have the descendent sequence *S*_2_.

We describe the type of problem we are confronted with according to a specific example, shown in Figure 5. Due to the 3-periodicity of the genetic code, there are three global reading frames in which a sequence may code in the forward direction, henceforth referred to as GRF1, GRF2 and GRF3. In viruses these reading frames tend to encode simultaneously for up to three different overlapping genes on each strand, resulting in multiple coding regions. We will be looking at single stranded RNA viruses, which predominantly code in the forward reading direction only. Amendments to our model would have to be made to include reverse reading frame encoded genes.

We are given two sequences *S*_1 _and *S*_2_, descended from a common ancestor, together with the gene structure *G *of *S*_1 _– in the case of Figure 5 this is a genome with two genes which overlap. Say these genes code in GRFs 1 and 2 respectively. Let us first assume we already have an alignment between our two sequences, and we wish to understand the way selection works on different regions of the genome. An initial question to ask would be, whether single and double coding regions behave in the same way. We thus, as shown, partition the genome into five segments, making a split wherever a gene starts or stops. These five segments we then assign to be of one of three region-types: non-, single- and double coding. When considering the effect a mutation of the indicated nucleotide *C *in the overlapping region of *S*_1 _might have, we must consider its coding role in both reading frames. In GRF1 it is in the third position of the codon AGC and in GRF2 in the second position of the codon GCT. In the genetic code the codon AGx codes for serine or argenine, depending on whether x is a purine or a pyrimidine, respectively. On the other hand GxT codes for four different amino acids, depending on the nature of x. Therefore a transition in the nucleotide C will have no effect on the amino acid encoded by GRF1, whereas a transversion will. In GRF2 on the other hand, both will result in a non-synonymous substitution. Additionally, the selection strengths acting on either gene might be different, due to one of them evolving faster than the other.

**Figure 6:**
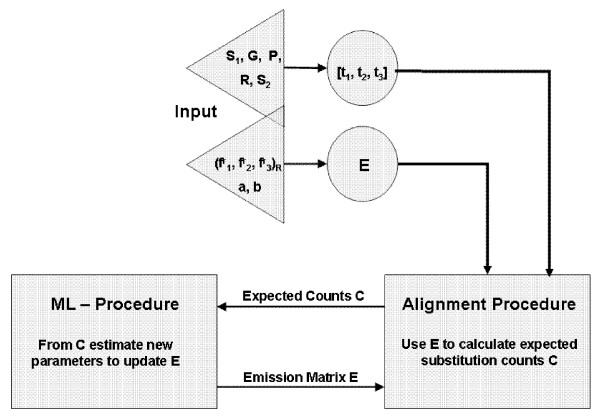
**Model Setup**. A graphic representation of our method. As input, we give the 'ancestral' sequence *S*_1_, its gene structure *G*, our desired partition *P *and our region annotation *R *of the partition segments. We also input the 'descendent' sequence *S*_2_, as well as our seed parameters for ${\left(f_{1}^{r},f_{2}^{r},f_{3}^{r}\right)}_{R}$, *a*, and *b*. From this we may generate both our seed emission matrix *E *and the type-annotation-array *t *= [*t*_1_, *t*_2_, *t*_3_] belonging to each locus along the sequence *S*_1_. These then get input into our alignment procedure, which subsequently over the sum of all possible alignments, calculates the expected counts *C *of a certain substitution of a certain type in a certain region. This information gets transferred to our maximum-likelihood (ML) method, which generates our new parameter values, maximizing the expected observations *C*. The resulting emission matrix *E *gets fed back into our alignment procedure, and the loop continues until a change in parameters is below some given threshold.

Since we wish to analyse selection happening over a reasonable evolutionary distance, our aim is to be able to draw conclusions without relying on a prior alignment. Instead of estimating evolutionary parameters using *observed *substitution counts froma fixed alignment, we will therefore use an alignment model to generate *expected *substitution counts and from these use a maximum likelihood method to estimate all evolutionary parameters. In this manner we may sum over the uncertainty of the alignment – an uncertainty that will be high for distantly related viruses. Since our alignment model includes a substitution model, we iteratively switch between both it and our ML-procedure. Figure 6 depicts the basic outline of our program.

### Substitution model

To be able to calculate the probability of a certain alignment between *S*_1 _and *S*_2_, we need to devise a model for the evolution of a sequence. We will be working with a simple 3-state HMM indel model, using a more complex nucleotide substitution model, given by an emission matrix *E*, for the emission probabilities. We wish to investigate region-specific selectional behaviour along the genome of *S*_1_. We may thus apply a partition *P *to our sequence *S*_1_, given by a sequence of partition points {*p*_0_, *p*_1_, ..., *p*_|*P*|_}, where clearly *p*_0 _= 0 and *p*_|*P*| _= |*S*_1_|. Because we are interested in certain global features, we may wish to group particular partition segments together into regions of a particular type. Say we have *R *regions, then each partition segment [*p*_*k*_, *p*_*k*+1_] with (0 ≤ *k *< |*P*|) gets assigned to a certain 'region-type' *r*, with (*r *≤ *R*), where regions of the same type are assumed to evolve in a similar way.

As stated above, since we are interested in investigating the evolutionary behaviour of viruses in particular, we wish to work with a substitution model, which specifically accounts for the presence of multiple coding regions. For our evolutionary model *E *we use a model very similar to the one in de Groot *et al*. [1]. For the convenience of those not familiar with our earlier work, we include a description of it in the following. Most amino acids are encoded by several different codons, meaning that a mutation may often result in no change in amino acid. Regarding a certain nucleotide in a codon, depending on its context, we may generally divide it into the following degeneracy classes, where a substitution would result in

• four times the same amino acid.

• two different amino acids, depending on whether a transition or transversion has occurred.

• four different amino acids, regardless of the type of substitution.

We shorthand these as being of type 4, 2 and 1 respectively. A few sites are not classifiable into one of the above three classes – ATx codes for three isoleucines and one methionine and CGG and GGG are synonymous although one results from the other by a transversion. Treating, for example, ATG as a type 1 site and ATA, ATC and ATT as type 4 sites, means however, that the approximations made by us are most likely to be minor.

We model the evolution of our sequences according to the Hein & Støvlbæk [5] model. When looking at a nucleotide in the ancestral sequence, for each reading frame we assign a certain state-dependent 'degeneracy-type' *t *to it, depending on its context. This will, in a coding region in a particular reading frame, be either of degeneracy 1, 2 or 4 and for non-coding will always be designated as 0. Since we are considering overlapping reading frames, we thus obtain for each nucleotide in the ancestral sequence a certain state-dependent 'degeneracy-type-array' *t *= [*t*1, *t*2, *t*3] – an array consisting of the degeneracy annotation of a nucleotide for each of the three reading frames. In our example in Figure 5 we can see an overlap between two genes, say genes *A *and *B*. This results in an annotation of [2, 1, 0] for our nucleotide C in the overlap, meaning that we have a degeneracy annotation of 2 and 1 with respect to gene *A *and *B *respectively.

Using this degeneracy annotation we incorporate the concept of selection factors into our framework: transitions and transversions occur according to the Kimura [8] model, and non-synonymous substitutions get accepted by a selection factor specified in the following.

Consider a nucleotide *x *in a region of type *r *in *S*_1 _with degeneracy-type array *t*. Then our factors will be given by *F*^*r *^[*t*1, *t*2, *t*3]_*ts *_and *F*^*r *^[*t*1, *t*2, *t*3]_*tv*_, for a transition and a transversion respectively. Within each region, we assign a selection factor to each gene within it, that is to say if gene *A *and gene *B *overlap in region *r*, we have selection factors $f_{A}^{r}$ and $f_{B}^{r}$ for mutations that result in a non-synonymous substitution in region *r *respectively in gene *A *and *B *only. In the case of a mutation causing a non-synonymous substitution in both genes, we would let it have selection factor $f_{AB}^{r}$. With our nucleotide of type [2, 1, 0], this would mean that a transition would be multiplied by the selection factor *F*^2 ^[2, 1, 0]_*ts *_= $f_{B}^{2}$, since it would result in a non-synonymous substitution only in the amino acid in region 2 in gene *B*. A transversion however would be multiplied by the selection factor *F*^2 ^[2, 1, 0]_*tv *_= $f_{AB}^{2}$ because it would cause a non-synonymous substitution in both gene *A *and gene *B*. If we were to assume independence between genes, the probability of a mutation causing a non-synonymous change in both genes would be given by *f*_*AB *_= *f*_*A*_·*f*_*B*_.

The probabilities of observing at a site of degeneracy [*t*1, *t*2, *t*3] in region *r *an identity, transition and transversion after time τ are given by exp **Q**^*r*^(*t*)τ where **Q**^*r*^(*t*) is the appropriate instantaneous Kimura rate matrix:

(1) $$P_{id}^{r}\left(\tilde{a},\tilde{b}\right)=1/4\cdot (1+\exp(-4\tilde{b})+2\exp(-2(\tilde{a}+\tilde{b})))$$

(2) $$P_{ts}^{r}\left(\tilde{a},\tilde{b}\right)=1/4\cdot (1+\exp(-4\tilde{b})-2\exp(-2(\tilde{a}+\tilde{b})))$$

(3) $$P_{tv}^{r}\left(\tilde{a},\tilde{b}\right)=1/2\cdot (1+\exp(-4\tilde{b}))$$

where

(4) $$\tilde{a}=a\cdot F^{r}{\left[t1,t2,t3\right]}_{ts}$$

(5) $$\tilde{b}=b\cdot F^{r}{\left[t1,t2,t3\right]}_{tv}$$

with *F *determined as explained above. We thus are able to construct an emission matrix *E*, where *E*(*r*, *t*1, *t*2, *t*3, *x*, *y*) is the probability of in region *r*, nucleotide *x *of type [*t*_1_, *t*_2_, *t*_3_] mutating into nucleotide *y*.

We would like to note that even though our alignment model does assume independence of sites, we model a local dependency in our evolutionary model by conditioning our emission probabilities on the nucleotide context in the ancestral sequence. An undoubtable improvement would be to model the dependency as continuous throughout the evolutionary process [17]. However, as noted by the authors themselves, the elaborate MCMC method developed in order to do this makes it a computationally intractable option.

### Alignment model

To compute the probability of an alignment we use a simple indel model with Match, Insert and Delete states. We have as alignment parameters a gap-opening, a gap-extension and a transition probability from any state to the end state. All other state transition probabilities may be derived from these. The Insert and Delete states emit a nucleotide from a uniform distribution, aligned to a gap. In the Match state nucleotide pairs are emitted according to our above model.

We wish to eliminate the bias in parameter estimation created by the use of a fixed alignment. For this, we work with a *probabilistic *alignment, which instead of producing an 'optimal' alignment, handles the set of all possible alignments and their likelihood. It computes posterior probabilities for each state at every nucleotide position. We thus are considering all possible sequence alignments and weighing them appropriately (see [23]), according to our indel model. This method has been previously used and described in further detail in [9,10]. Note, that when referring to the insertion and deletion states, the related posteriors are added together so that we obtain the posterior probability of a certain nucleotide not being aligned, as opposed to belonging to a particular gap.

During the alignment procedure, our alignment parameters are estimated in a few iterations of the Baum-Welch algorithm [3]. The implementation of the algorithm, including banding to cut computational demands, was generated automatically by the HMM compiler program HMMoC [11].

### Full model

As shown in Figure 6, we initially have as an input all the sequence and genome structure data, as well as a set of seed parameters. We subsequently use our alignment model to generate the posterior probabilities of every nucleotide position being in each state. This is done by using the forward and backward algorithms applied to our alignment indel model, as is standard HMM procedure. From these posteriors we may calculate, for each degeneracy in each region, the *expected *number of times an identity, transition and transversion is used. For a site of degeneracy *t *= [*t*_1_, *t*_2_, *t*_3_] in region *r*, let this be $x_{id,t}^{r}$, $x_{ts,t}^{r}$ and $x_{tv,t}^{r}$ respectively. Since $P_{id,t}^{r}$, $P_{ts,t}^{r}$ and $P_{tv,t}^{r}$ were the probabilities for a site of degeneracy *t *in region *r *of an identity, transition or transversion occurring (see equations 1, 2, 3), we may rewrite the emission term of the log likelihood log *L *as follows:

(6) $$\log L=\sum_{t}\sum_{r}x_{id,t}^{r}\log P_{id,t}^{r}+x_{ts,t}^{r}\log P_{ts,t}^{r}+x_{tv,t}^{r}\log P_{tv,t}^{r}$$

For this function of the 3*R *+ 2 emission parameters ${\left(f_{1}^{r},f_{2}^{r},f_{3}^{r}\right)}_{R}$, *a *and *b *we now find the maximum likelihood estimates using the Newton-Raphson iteration method. We may do this by taking the explicit derivatives of the likelihood function, possible because of the simple substitution model used. If we had opted for a more complicated model, we would need optimization methods that did not rely on derivatives and would subsequently be slower, though the estimation would still be possible. Once the change in log-likelihood between two iterations has fallen below some given threshold, we take the likelihood to have converged. We then generate a new emission matrix *E *to be fed back into our alignment procedure in order to generate new posterior probabilities.

Once the likelihood function has converged below some set threshold, we output the final set of estimated selection parameters. We may also, if desired, construct an alignment, either using the Viterbi path or posterior decoding.

### Extension to Multiple Sequences

We would like to be able to apply our method to multiple sequences, thus extrapolating more information where possible. We could of course devise a multiple alignment indel model, and develop a new likelihood function from which to maximize over all tree branches simultaneously. This however would be of computational much higher demand, runtime increasing exponentially with the addition of each new sequence. Instead, we therefore opt to work with a multiple pairwise alignment under the assumption of a star shaped tree, with the reference sequence as the root in the star topology. This implies viewing the evolution of the pairwise sequences as independent, which is an approximation which we wish to address in future work. This merely requires per additional sequence an extra transition and transversion parameter, since selection is acting on the gene in the ancestor and we assume this to be constant over all branches. The modification to our program is thus trivial, with only a linear increase in runtime.

As an input we have, for lack of better terminology, the ancestral sequence *A *and its *N *descendants *D*_1_, ..., *D*_*N*_, together with the seed parameters for the selection factors (*f*_1_, *f*_2_, *f*_3_)_*R *_on each region *R *as well as *n *transition and transversion parameters (*a*, *b*)_*n *_respectively.

We then build a set of *N *pairwise alignments between the ancestor *A *and its *N *descendants. Each one of these obtains a likelihood function log *L *as given in equation 6. Now we create a new likelihood function log L* which is the sum of the *N *log likelihoods. If $x_{id,t}^{n,r}$ is the number of expected identities of type *t *in region *r *between the ancestral sequence *A *and its *n*^*th *^descendant, then our assumption of independence implies that

(7) $$\log L*=\sum_{n}\sum_{t}\sum_{r}x_{id,t}^{n,r}\log P_{id,t}^{n,r}+x_{ts,t}^{n,r}\log P_{ts,t}^{n,r}+x_{tv,t}^{n,r}\log P_{tv,t}^{n,r}$$

is the full likelihood of observing all *N *sequences under our model. Note here, that the probabilities *P *are dependent on the sequence-dependent transition and transversion rates (*a*, *b*)_*n *_and the selection factors (*f*_1_, *f*_2_, *f*_3_)_*R *_which in turn are *not *dependent on *n*, since we are assuming selection to occur on the gene in the ancestral sequence.

Maximizing this new log likelihood function, we proceed as above and estimate a new set of selection factors and a set of sequence specific transition and transversion rates, from which we may generate a new set of pairwise statistical alignments.
